# Sexual reproduction and the polygenic architecture of azole resistance in agricultural populations of *Aspergillus fumigatus*

**DOI:** 10.3389/fmicb.2026.1843477

**Published:** 2026-06-03

**Authors:** Jiarui Huang, Haicheng Liu, Yu He, Ying Zhang

**Affiliations:** 1 State Key Laboratory for Conservation and Utilization of Bio-Resources in Yunnan, Yunnan University, Kunming, Yunnan, China; 2 Yunnan Key Laboratory of Basic Research and Innovative Application for Green Biological Production, Yunnan University, Kunming, Yunnan, China

**Keywords:** adaptive evolution, antimicrobials, greenhouse populations, quantitative trait, sexual fitness

## Abstract

**Introduction:**

Agricultural environments, particularly greenhouses, are recognized as hotspots for the evolution of azole resistance in the opportunistic pathogen Aspergillus fumigatus. This study aimed to investigate the role of sexual reproduction and its underlying genetic architecture in driving resistance in Yunnan Province, China.

**Methods:**

We integrated large-scale sexual crossing experiments with hierarchical genetic association analysis. Sexual fitness was compared between greenhouse and outdoor populations. A genome-wide association study (GWAS) on greenhouse strains identified candidate SNPs, which were then analyzed via hierarchical association mapping in 300 sexual progeny strains to decipher the genetic architecture of resistance.

**Results:**

Greenhouse populations exhibited significantly higher sexual fitness, evidenced by greater mating type diversity, mating success (28.36% vs. 12.38%), and ascospore viability. Resistance was shown to be a complex quantitative trait primarily governed by epistatic interactions, where the strength of genetic associations increased with SNP combination complexity. A key, previously uncharacterized locus (SNP6) consistently showed significant effects. Comparative analysis revealed divergent genetic architectures for itraconazole (ITR) and voriconazole (VOR) resistance. Functional annotations implicated SNPs in oxidative stress response (e.g., salicylate hydroxylase) and cell wall integrity (e.g., chitin synthase) pathways.

**Discussion:**

We propose a synergistic model wherein high sexual competency provides a platform for recombination, acting upon a standing epistatic landscape to facilitate rapid adaptive evolution. Our findings highlight the potential for resistance allele spread via sexual reproduction and suggest that resistance surveillance may need to account for multi-locus genotypes.

## 1 Introduction

*Aspergillus fumigatus* Fresen., 1863 is a ubiquitous filamentous fungus that thrives in soil, water, and air, and is the leading cause of aspergillosis in humans. This opportunistic pathogen can trigger a wide spectrum of clinical manifestations ranging from allergic bronchopulmonary aspergillosis to invasive aspergillosis (IA). In immunocompromised patients, IA is associated with mortality rates exceeding 50%–90%, making *A. fumigatus* one of the most life-threatening fungal pathogens worldwide (Machado et al., 2024).

Triazoles—including itraconazole, voriconazole, and posaconazole—are the frontline agents for aspergillosis treatment (Rehman and Long, 2024). However, the global emergence of azole-resistant *A. fumigatus* (AR*Af*) has drastically reduced therapeutic efficacy and complicated clinical management, with resistance rates increasing in both clinical and environmental settings (Chowdhary et al., 2013; Tashiro et al., 2025; Rhodes et al., 2022). Thus, understanding the origins, mechanisms, and transmission of antifungal resistance in *A. fumigatus* has become a public health priority.

The predominant mechanism of azole resistance is mutation in the *cyp51A* gene, which encodes the azole drug target 14α-sterol demethylase. Common resistance-associated alleles include point mutations such as TR_34_/L98H and TR_46_/Y121F/T289A (Lu et al., 2025; European Food Safety Authority (EFSA), European Centre for Disease Prevention and Control (ECDC), European Chemicals Agency (ECHA), European Environment Agency (EEA), European Medicines Agency (EMA), European Commission's Joint Research Centre (JRC), 2025). However, not all resistant isolates harbor *cyp51A* alterations, suggesting additional genetic determinants remain undiscovered (Sharma et al., 2019; Fan et al., 2021; Lehmann et al., 2025). Other known mechanisms include overexpression of *cyp51B*, mutations in transcriptional regulators (*hapE*), or metabolic genes (*hmg1*) (Souza et al., 2023; Rybak et al., 2019).

Environmental surveys highlight the complexity of resistance evolution. For example, Zhou et al. reported that ~80% of greenhouse-derived isolates from Yunnan, China, were resistant to itraconazole or voriconazole, yet over 70% of these resistant strains lacked canonical *cyp51A* mutations (Zhou et al., 2021). These findings suggest that azole resistance in *A. fumigatus* is polygenic and may involve novel loci or interactions between mutations. Genome-wide association studies (GWAS) have begun to uncover new single-nucleotide polymorphisms (SNPs) associated with azole or amphotericin B resistance, but functional validation remains limited (Fan et al., 2021; Zhao et al., 2021).

The sexual cycle of *A. fumigatus* was only discovered in 2009 (O'Gorman et al., 2009). It is controlled by the mating-type (MAT) locus, which exists as two idiomorphs, MAT1-1 and MAT1-2. Successful mating requires strains of opposite MAT types. Global surveys indicate that *A. fumigatus* populations maintain a near 1:1 ratio of mating types (Paoletti et al., 2005), consistent with active sexual reproduction in nature. Laboratory studies have demonstrated the formation of cleistothecia and viable ascospores, with recombination frequencies among the highest reported in filamentous fungi (Lofgren et al., 2022; Auxier et al., 2023).

Sexual reproduction in *A. fumigatus* serves as a key evolutionary driver for the origin and spread of antifungal resistance by facilitating genetic recombination, which shuffles single-nucleotide polymorphisms (SNPs) across progeny populations, leading to novel genotypic combinations that may confer resistance phenotypes (Ashton et al., 2022). Furthermore, hybridization experiments show that resistant alleles can be transmitted between genetically distinct parental strains, producing offspring with new resistance and virulence profiles (Korfanty et al., 2021). For instance, bulk segregant analysis (BSA) combined with experimental crosses has been employed to identify SNPs associated with azole resistance; through backcrossing resistant and sensitive strains, researchers narrowed down genomic regions harboring candidate genes (e.g., *cyp51A* mutations) that were recurrently selected in progeny pools, demonstrating how sexual reproduction amplifies resistance alleles in natural populations (Ashton et al., 2022). The frequency of such SNPs in sexual offspring—quantified via high-throughput sequencing—correlates with quantitative traits like minimum inhibitory concentration (MIC), highlighting the polygenic basis of resistance and the role of sexual recombination in generating phenotypic diversity (Fan et al., 2021). However, the extent to which this occurs in highly resistant environmental hotspots remains unclear.

Moreover, integrative studies leveraging genome-wide association analyses (GWAS) and progeny validation reveal that SNP-SNP interactions significantly influence growth phenotypes under drug stress. For example, in crosses between amphotericin B-resistant isolates, specific SNP combinations [e.g., intergenic variants on chromosomes 5 and 6 resulted in transgressive phenotypes in progeny, such as enhanced growth at sub-MIC concentrations, underscoring the SNP–SNP interactions shaping resistance evolution (Fan et al., 2021)]. This aligns with findings on phenotypic heterogeneity, where sexual reproduction contributes to continuous trait distributions (e.g., tolerance levels) through non-genetic mechanisms like gene expression noise, further complicating resistance dynamics (Kozubowski and Berman, 2025). These observations suggest that sexual recombination may be a major driver of resistance evolution in natural populations, but the extent to which this occurs in highly resistant environmental hotspots remains unclear. Key unanswered questions include: To what extent does active sexual reproduction contribute to resistance development in these hotspots? How do SNP-SNP interactions in recombinant progeny influence quantitative resistance phenotypes? And what methodological pathways—such as integrated GWAS-BSA approaches coupled with progeny validation—best elucidate the genetic architecture of resistance in these settings? Addressing these questions requires focusing specifically on greenhouse populations, employing controlled crosses to quantify recombination rates, and applying SNP-based mapping to unravel the complex genetic interactions driving resistance emergence in these critical environmental reservoirs.

Southwest China, particularly Yunnan Province, is one of the most biodiverse regions globally, but it also represents a unique ecological niche for resistance evolution. Intensive fungicide use in agricultural greenhouses has created strong selective pressure for resistant *A. fumigatus*. Surveys across Yunnan revealed that ~80% of greenhouse isolates were resistant to triazoles, the highest frequency reported worldwide, compared to ~20% resistance in open-field soils (Zhou et al., 2021; Zhou et al., 2022). Greenhouse soils thus serve as ecological “hotbeds” for resistance development and may represent a critical source for clinical AR*Af* infections. Despite extensive surveys of resistance prevalence, little is known about the sexual reproductive potential of greenhouse-derived populations or the genetic mechanisms underlying their extreme resistance levels. Specifically, it remains unclear whether sexual reproduction in these populations contributes to the recombination and dissemination of resistance alleles. Understanding these dynamics is crucial for predicting the future trajectory of antifungal resistance and its potential spillover from agricultural to clinical settings.

In this study, we investigated the role of sexual reproduction in the evolution of azole resistance in *A. fumigatus* populations from greenhouse and outdoor environments in Yunnan Province. To systematically unravel this process, we addressed three questions: (i) Do greenhouse populations, which are under strong antifungal selection, retain a functionally efficient sexual cycle, and what is the viability of their sexual progeny (ascospores)? (ii) How do genetic and geographic distances influence mating success and reproductive fitness across diverse environmental isolates, thereby shaping the potential landscape for gene flow? Given the confirmed potential for sexual recombination, we then asked: (iii) Which novel genetic variants are associated with azole resistance, and how do they—individually and through interactions—shape the resistance phenotypes in the resulting sexually recombinant offspring? To answer these questions, we first conducted large-scale crossing experiments, quantifying cleistothecia formation and ascospore germination, and analyzing their correlation with genetic and geographic distance. Subsequently, to directly trace the fate of resistance alleles during sexual reproduction, we integrated genome-wide association analysis (GWAS) with experimental validation in hybrid progeny using PCR-RFLP genotyping and hierarchical antifungal susceptibility testing. This combined approach allowed us to dissect the contribution of candidate SNPs and their interactions to the azole resistance phenotype.

## 2 Materials and methods

### 2.1 Fungal isolates, antifungal susceptibility testing and phenotype categorization

We assessed 226 *A. fumigatus* isolates collected from greenhouse soils in Yunnan, China (Zhou et al., 2022). Based on their greenhouse of origin, the strains were divided into nine distinct populations, designated as Populations 1 through 9. Antifungal susceptibility to itraconazole (ITR), voriconazole (VOR), was determined according to CLSI M38-A2 guidelines, using established clinical breakpoints to define resistance. The MIC was defined as the lowest drug concentration that resulted in 100% visual inhibition of fungal growth compared to the drug-free control. Based on the CLSI clinical breakpoint, isolates with an MIC ≥ 4 mg/L for either azole were categorized as “resistant” (R), while those with an MIC < 4 mg/L were categorized as “susceptible” (S). This binary phenotype was used for all subsequent association analyses.

### 2.2 Sexual crossing assay and analysis of geographic and genetic distance effects

To evaluate sexual compatibility and the potential for genetic exchange across ecological boundaries, we performed large-scale crossing experiments. The experimental design included strains from both greenhouse and outdoor agricultural environments to specifically test for pre-zygotic reproductive barriers, assess the potential for gene flow of adaptive alleles (e.g., azole resistance), and analyze the influence of geographic and genetic distance on mating success. A total of 113 strains were used in mating experiments: 47 greenhouse isolates, 65 outdoor environmental isolates (Zhou et al., 2022), and the reference strain Af293. From the 47 greenhouse isolates, 5 were selected as “tester” strains. These 5 greenhouse tester strains were then crossed with the 65 outdoor isolates and Af293, forming a distinct set of 71 strains for inter-population/parental background crosses (Table 1; Supplementary Table S1). Each strain was crossed in all possible combinations with strains of the opposite mating type, along with all self-crossed strains serving as controls, generating 1,928 unique mating pairs. This comprehensive crossing matrix allowed us to quantify mating success (cleistothecia formation) and subsequently analyze its correlation with both the geographic distance (km) and the genetic distance (based on STR profiles) between parental strains within and between the two environments.

**Table 1:** Crossing scheme of this study.

**Group**	Strain Source	Numbers	MAT1-1	MAT1-2	No. of cross pairs
1	Greenhouse only (excluding 5 selected strains)	47	25	22	550
2	Mixed population 5 selected Greenhouse + 65 Outdoor + Af293	71	35	36	1,260
3	Selfing (all unique strains)	113	—	—	118
Total	Overall	113	—	—	1928

### 2.3 Whole-genome sequencing, SNP calling, and initial filtering

Genomic DNA was extracted from all 226 isolates. Whole-genome sequencing was performed on the Illumina NovaSeq 6000 platform. The raw sequencing reads were quality-trimmed and aligned to the *A. fumigatus* Af293 reference genome (Assembly ASM265v1). Initial variant calling identified a comprehensive set of genetic polymorphisms. From this set, a high-quality SNP dataset was generated by applying strict filters for read depth (>10), genotype quality (>30), and call rate (>90%). Raw FASTQ files for the isolates sequenced in this study were uploaded to the NCBI Sequence Read Archive (SRP637305) and are publicly available under BioProject PRJNA1345449.

### 2.4 Linkage disequilibrium pruning and selection of tag SNPs

To minimize the inclusion of redundant SNPs in high linkage disequilibrium (LD), the initial SNPs were pruned using an LD-based clumping procedure. A pairwise *r*^2^ threshold of 0.8 was applied within a 100 kb window. This step, a standard procedure in genome-wide association studies (GWAS), refined the initial SNP set to tag SNPs that were largely independent of each other, representing distinct genomic regions for association testing.

### 2.5 Genome-wide association analysis

A genome-wide association analysis was performed to test for statistical associations between the tag SNPs and the binary azole resistance phenotype. A Fisher’s exact test was implemented for each SNP, comparing the allele frequencies between the resistant and susceptible groups. A *p*-value of less than 0.05 was considered statistically significant for this discovery phase. The analysis identified a subset of SNPs significantly associated with ITR resistance, VOR resistance, or both.

### 2.6 Selection of candidate SNPs for progeny genotyping

From the tag SNPs identified through the GWAS, a final set of candidate SNPs was selected for downstream genotyping in the sexual progeny population. The selection criteria were twofold: (i) Statistical Significance: SNPs showing a significant association (*p* < 0.05) with resistance to one or both azoles were prioritized; (ii) Genotyping Feasibility: To enable cost-effective and high-throughput genotyping of the large progeny population, SNPs that created or disrupted a restriction enzyme recognition site were preferentially selected, allowing for the use of PCR-RFLP (Polymerase Chain Reaction - Restriction Fragment Length Polymorphism) analysis, the primer sequences for the seven SNP loci are summarized in Supplementary Table S2.

### 2.7 Sexual crosses and progeny population

Three pairs of sexually compatible greenhouse strains with varying azole resistance profiles were crossed on oatmeal agar plates. After 3–4 months of incubation in the dark at 25 °C, cleistothecia were harvested, and ascospores were isolated. A total of 300 progeny strains (100 from each cross) were randomly selected for further analysis. The MICs of ITR and VOR for all progeny were determined as described in section 2.1.

All six parental strains used in the genetic crossing were confirmed to be wild-type for the c*yp51A* gene, harboring none of the known resistance-associated mutations (e.g., TR_34_/L98H). The *cyp51A* status of the 300 progeny was not assessed, as the focus of this study was on mapping novel loci independent of the canonical *cyp51A*-mediated resistance mechanism.

### 2.8 Genotyping of progeny and growth phenotyping

The genotypes of the 300 progeny strains at the candidate SNP loci were determined using PCR-RFLP. The growth of each progeny strain was also quantified on SDA plates containing a gradient of ITR and VOR concentrations (e.g., 0.25, 0.5, 1.0, 2.0, 4.0 mg/L, 8.0 mg/L, and 16.0 mg/L). The growth rate was calculated as the colony diameter under drug pressure relative to the diameter on a drug-free control plate after a standard incubation period.

## 3 Results

### 3.1 Mating type distribution and large-scale sexual crossing in *Aspergillus fumigatus*

The investigation into the reproductive potential of *A. fumigatus* populations from greenhouse and outdoor environments revealed a nearly 1:1 ratio of mating types. Specifically, among the greenhouse isolates, the proportions of MAT1-1 and MAT1-2 were 45.78% and 54.22%, respectively. This balanced distribution is consistent with populations undergoing sexual reproduction.

Based on antifungal resistance profiles and mating types, we designed a comprehensive set of sexual crosses. A total of 47 greenhouse-derived strains, including the control strain Af293 (MAT1-2), were selected (Zhou et al., 2021). This collection comprised 25 MAT1-1 and 22 MAT1-2 strains (Supplementary Table S1). Phylogenetic analysis based on short tandem repeat (STR) genotyping further supported the potential for genetic exchange, as strains of opposite mating types were evenly interspersed across the evolutionary tree rather than clustering by mating type, indicating a lack of clonal population structure and suggesting historical recombination (Supplementary Figure S1A). Each of the 47 strains was paired with every strain of the opposite mating type, resulting in 25 × 22 = 550 unique hybrid combinations. To serve as a negative control, all 47 strains were also subjected to self-crossing. In total, this arm of the study encompassed 550 hybrid pairs plus 47 self-crosses, amounting to 597 distinct crossing experiments.

Initially, 65 outdoor isolates were selected (Zhou et al., 2022). Furthermore, to specifically test the mating ability between greenhouse and outdoor populations, 5 additional greenhouse strains were chosen for inter-population crosses. Including the Af293 control, this resulted in a final set of 71 strains (Supplementary Table S1). Phylogenetic analysis of these 71 strains also showed an even distribution of the 35 MAT1-1 and 36 MAT1-2 isolates across the tree (Supplementary Figure S1B). Each strain was crossed with all strains of the opposite mating type, generating 35 × 36 = 1,260 hybrid combinations. All 71 strains were also self-crossed as negative controls, bringing the total number of crossing experiments in this environmental set to 1,260 + 71 = 1,331.

The set of greenhouse strains generated 550 unique crosses, resulting in a significantly higher mating success rate (28.36%, 156/550) compared to that of outdoor strains (12.38%, 156/1,260). This indicates that greenhouse strains possess a greater inherent capacity for sexual reproduction under laboratory conditions. As expected, none of the self-cross controls produced cleistothecia, confirming true sexual reproduction requires compatible mating types. The number of cleistothecia produced ranged from fewer than 4 to more than 100 per mating pair, indicating significant genetic or physiological influences on the efficiency of the sexual cycle in *A. fumigatus*.

**Figure 1:**
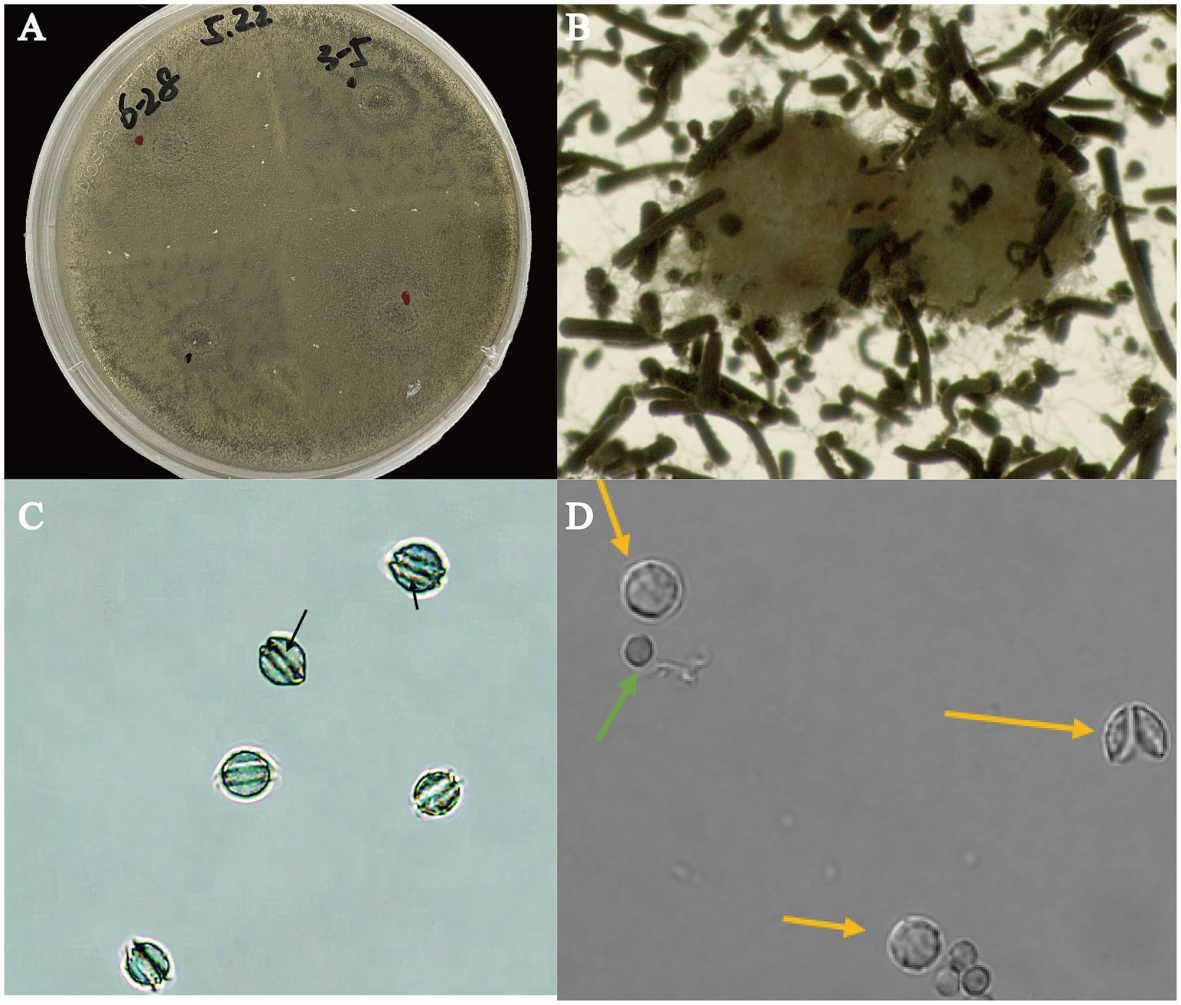
Sexual structures and progeny of *Aspergillus fumigatus* from controlled crosses. **(A)** Schematic overview of the sexual reproduction process leading to ascospore formation. **(B)** Two mature cleistothecia formed at the junction zone of two compatible strains on oatmeal agar. **(C)** Released ascospores showing the characteristic equatorial ridges (black arrows). **(D)** A mixed population of ascospores (yellow arrows) and smaller, asexual conidia (green arrow).

Mating success varied substantially among greenhouse populations. Pop 4 in the greenhouse exhibited the highest success rate (42.1%), followed by Pop 5 (36.8%), Pop 2 (31.7%), and Pop 1 (29.2%), whereas Pop 6 (23.7%) and Pop 7 (25.4%) were lowest (Supplementary Table S3). The productivity of successful crosses, measured by the number of cleistothecia produced, also varied considerably (Figures 1A,B). Morphological examination revealed typical *A. fumigatus* cleistothecia containing mature ascospores (Figures 1C,D). While most successful pairs produced few cleistothecia (1-10), a subset of crosses, particularly those involving strains from Greenhouses 3, 4, 5, 7, 8, and 9, were highly productive, yielding between 51 and 100 cleistothecia. This indicates that specific genetic combinations can result in high fertility: ~20% producing 11–20, and ~5% producing >50 (Figure 1B).

Cleistothecia formation, indicating successful sexual reproduction, was observed in 156 pairs of large-scale crossing assay involving 71 greenhouse and outdoor strains, resulting in an overall mating success rate of 12.38% (156/1,260). None of the 71 strains were universally compatible, able to mate successfully with all strains of the opposite mating type. The strain LGH3 (MAT1-1) exhibited the highest individual mating success, forming cleistothecia with 16 out of 36 tested partners (44.44%). Comparative analysis revealed that mating success rates between strains from different geographic populations were generally higher than rates within the same population, although this difference was not statistically significant (inter-population: 12.55%, 148/1179; intra-population: 9.88%, 8/81; *p* = 0.6013 by Fisher’s exact test) (Supplementary Table S3). Notably, strains from the Ninglang population showed a significantly different mating success rate compared to each of the other 13 populations. Overall, the mating success rates observed for outdoor environmental strains, both within and between populations, were lower than those previously recorded for greenhouse-derived strains.

Because sexual reproduction occurred with measurable but variable success, we next investigated whether mating outcomes were influenced by geographic or genetic distances between parents. Pairings spanned geographic distances of 0.5–5 km between collection sites. Neither geographic distance nor genetic distance (based on STR profiles) showed significant correlation with mating success (Fisher’s exact test, *p* > 0.05) (Supplementary Figures S2A–D). Similarly, cleistothecia counts (Kruskal–Wallis test, *p* = 0.59 and 0.78 for geographic and genetic distance, respectively) (Supplementary Figures S2E–H). These results suggested that mating compatibility is largely unrestricted, raising the question of whether the sexual progeny was viable and capable of germination.

In contrast, for the widely dispersed outdoor environmental strains (0–658 km apart), a more complex pattern emerged. While there was no significant overall correlation between geographic distance and mating success, in pairwise comparisons between groups across different geographical distance ranges (Supplementary Figure S2C,D), the mating success rate of strains within the 492–574 km range showed statistically significant differences compared to the rates of strains within the 0–82 km, 82–164 km, 164–246 km, and 328–410 km ranges (*p* = 0.0131, *p* = 0.0282, *p* = 0.0162, and *p* = 0.0096, respectively) (Supplementary Figure S2L). This points to the existence of isolation-by-distance, where geographical barriers in the mountainous terrain of Yunnan likely restrict gene flow, reducing sexual compatibility between the most distant populations.

The viability of the sexual progeny was assessed by measuring ascospore germination. Among the 156 successful crosses with purely greenhouse strains, 141 (90.4%) yielded viable ascospores (Supplementary Table S4). Germination rates ranged from 0.3 to 47.1%, with a median of 16.8%. Population-level differences were apparent: Pop 2 achieved the highest proportion of viable crosses (97.1%), followed by Pop 4 (94.3%), Pop 3 (92.6%), and Pop 1 (89.5%), whereas Pop 7 was lowest (85.2%). A high percentage of successful crosses produced viable ascospores: 90.38% (141/156) for greenhouse crosses and 84.62% (132/156) for outdoor strain crosses. However, the germination rates themselves were generally low, ranging from 0.28% to 47.25%, with greenhouse crosses showing a significantly higher average germination rate (9.88%) than outdoor strain crosses (7.07%). This suggests that not only is mating initiation more likely between greenhouse strains, but the resulting sexual spores also exhibit greater viability.

Sexual reproductive fitness revealed two critical components: the initial formation of cleistothecia (pre-zygotic success) and the subsequent viability of ascospores (post-zygotic success). For strain pairs that formed cleistothecia, the viability of their ascospores represents the second key feature of their sexual reproductive fitness. A high rate of ascospore germination signifies high post-zygotic reproductive fitness, while a low germination rate indicates the presence of a post-zygotic reproductive barrier. A strong positive correlation was observed between a strain’s overall mating success (number of successful crosses) and its contribution to crosses producing viable ascospores. In the greenhouse population, highly sexually compatible strains like 3–5 (MAT1-1) and 4–20 (MAT1-2) not only had the highest number of successful crosses but also contributed the most to the pool of viable ascospores. Conversely, strains with very few successful crosses, such as 3–14 and 6–28, produced minimal viable offspring. This trend was mirrored in the outdoor strains, where the most prolific individuals, LGH3 (MAT1-1) and LGH19 (MAT1-2) from the Ninglang Lugu Lake population, generated the highest number of viable ascospore-producing crosses. In contrast, strains from populations like Guangnan and Longyang that had very low mating success also showed minimal ascospore viability. Overall, greenhouse strains demonstrated a higher overall frequency of successful crosses yielding viable ascospores compared to the outdoor strains. This indicates that the enhanced sexual competency of greenhouse populations encompasses both pre-zygotic compatibility and post-zygotic success, resulting in a higher overall reproductive fitness, which may accelerate adaptive evolution in these agricultural environments.

Similar to the mating success results, geographic and genetic distance had no significant impact on ascospore germination rates within the greenhouse populations (Mantel test, *p* > 0.05) (Supplementary Figures S2I–L). For the outdoor strains, while no significant overall correlation was found, the germination rate for crosses from the Guangnan population was significantly lower than that of several other populations, indicating potential local adaptation or genetic factors affecting spore viability (Supplementary Table S4).

### 3.2 Selection of candidate SNPs from genome-wide association analysis

The comprehensive analysis of sexual reproduction established that greenhouse *A. fumigatus* populations exhibit superior overall fitness, characterized by higher mating success, cleistothecia production, and ascospore viability compared to outdoor strains, with little influence from geographic or genetic distance at the population level. This robust sexual competency, particularly within the greenhouse environment, provided an ideal genetic background for a more targeted investigation. Furthermore, 70% of highly resistant strains in the greenhouses lacked canonical *cyp51A* mutations, emphasizing that resistance is shaped by multiple mechanisms beyond the well-characterized TR_34_/L98H or TR_46_/Y121F/T289A alleles (Zhou et al., 2021).

To dissect the genetic underpinnings of the continuous variation in azole resistance as a key quantitative trait, we narrowed our focus to the progeny derived from controlled sexual crosses. Specifically, we utilized three sexually compatible pairs of greenhouse strains to generate segregating offspring populations. This designed approach allowed us to move beyond population-level phenotypes and precisely track the segregation and interaction of seven specific SNP loci in the meiotic progeny. The primary objective was to determine how the recombination of these alleles during sexual reproduction shapes the quantitative resistance landscape, thereby explicitly probing the epistatic interactions between these critical resistance loci.

To identify genetic variants associated with azole resistance in *A. fumigatus*, we performed a genome-wide association study (GWAS) based on whole-genome sequencing data from 226 greenhouse strains collected from Jinning, Yunnan Province. Initial analysis identified 545 high-quality SNPs, which were subsequently pruned to remove variants in high linkage disequilibrium (LD). LD analysis revealed 22 SNPs with significant linkage, which were selected for further investigation (Table 2).

**Table 2:** Characteristics and association statistics of 22 candidate SNPs identified through linkage disequilibrium analysis.

Chromosome	Location	Gene ID	Predictive effect	Note	Mutant	ITR (*p*)	VOR (*p*)
**NC-007195.1**	**3,487,309**	***AFUA-2G13470***	**Encoding**	**Pyrimidine 5′-nucleotidase, putative**	**T to C**	**0.0001**	**0**
**NC-007197.1**	**3,752,686**	***AFUA-4G14300***	**Non-coding**	**Dynamin family GTPase, putative**	**C to T**	**0.0272**	**0.1871**
**NC-007194.1**	**279,356**	***AFUA-1G00770***	**Non-coding**	**Hypothetical protein**	**C to T**	**0.4203**	**0.0165**
**NC-007200.1**	**278,750**	***AFUA-7G01050***	**Encoding**	**Salicylate hydroxylase, putative**	**G to C**	**0.1989**	**0.0938**
**NC-007200.1**	**289,774**	***AFUA-7G01102***	**Encoding**	**Hypothetical protein**	**C to T**	**0.8554**	**0.6318**
**NC-007201.1**	**70,735**	***AFUA-8G00342***	**Non-coding**	**Hypothetical protein,hypothetical protein**	**G to A**	**0.3806**	**0.0785**
**NC-007195.1**	**3,469,525**	***AFUA-2G13440***	**Encoding**	**Chitin synthase ChsE**	**A to G**	**0.0005**	**0.0002**
NC-007194.1	279,314	*AFUA-1G00770*	Non-coding	Hypothetical protein	G to A	0.852	0.0027
NC-007195.1	1,073,254	*AFUA-2G03960*	Non-coding	Acid phosphatase	A to T	0.0333	0.118
NC-007195.1	1,073,260	*AFUA-2G03960*	Non-coding	Acid phosphatase	A to C	0.0147	0.0888
NC-007195.1	3,548,935	*AFUA-2G13640*	Non-coding	Serine/threonin protein kinase, putative	T to C	0.0003	0.00001
NC-007197.1	2,762,423	*AFUA-4G10570*	Non-coding	Cell cycle regulatory protein, putative	A to C	0.433	0.0018
NC-007197.1	2,762,487	*AFUA-4G10570*	Non-coding	Cell cycle regulatory protein, putative	A to G	1	0.0106
NC-007197.1	2,766,792	*AFUA-4G10600*	Encoding	GTPase activating protein (Gyp5), putative	C to A	0.4147	0.0071
NC-007197.1	2,767,284	*AFUA-4G10600*	Non-coding	GTPase activating protein (Gyp6), putative	T to C	0.3164	0.0132
NC-007197.1	2,767,611	*AFUA-4G10600*	Non-coding	GTPase activating protein (Gyp7), putative	T to G	1	0.0313
NC-007197.1	2,767,674	*AFUA-4G10600*	Non-coding	GTPase activating protein (Gyp8), putative	T to C	1	0.0106
NC-007199.1	24,610	*AFUA-6G00110*	Non-coding	Dihydrodipicolinate synthetase family protein	A to G	0.0002	0.00001
NC-007199.1	24,621	*AFUA-6G00110*	Non-coding	Dihydrodipicolinate synthetase family protein	A to G	0.0001	0.00001
NC-007199.1	3,766,475	*AFUA-6G14670*	Non-coding	Hypothetical protein	T to C	0.0001	0.00001
NC-007200.1	1,964,663	*AFUA-7G08490*	Encoding	Cass V chitinase, putative	A to T	0.147	0.8876
NC-007200.1	1,984,541	*AFUA-7G08530*	Non-coding	hypothetical protein	A to G	0.3286	0.225

The first seven bolded loci are SNP1 to SNP7.

The primary criterion was the strength of association with the resistance phenotype. Fisher’s exact test was employed to assess the association between these 22 high-LD SNPs and azole resistance phenotypes, using clinical breakpoints (MIC ≥ 4 mg/L) according to CLSI M38-A2 guidelines. The analysis revealed that 9 SNPs were significantly associated with ITR resistance and 14 with VOR resistance (*p* < 0.05). Notably, 6 SNPs showed significant associations with resistance to both azoles, indicating potential pleiotropic effects, while 11 were associated with either ITR or VOR resistance alone. In total, 17 of the 22 SNPs (77.3%) were significantly correlated with azole resistance in *A. fumigatus* (Table 2). We further selected SNPs based on the feasibility of genotyping a large progeny population using a cost-effective method. Specifically, we prioritized SNPs that either created or disrupted a restriction enzyme recognition site, making them amenable to PCR-RFLP analysis. This dual-filtering strategy resulted in the selection of seven high-priority candidate SNPs, designated SNP1 to SNP7.

Notably, six of the seven SNPs (all except SNP3) showed a highly significant association with ITR resistance (*p* < 0.0005). Similarly, six SNPs (all except SNP2) were significantly associated with VOR resistance, with four of them (SNP1, SNP4, SNP5, SNP6) exhibiting extreme significance (*p* < 0.00001). The fact that the majority of our selected candidates fell into this category confirms that our bioinformatic pipeline successfully enriched for SNPs with the strongest genetic effects. Notably, three of these (SNP4, SNP5, SNP7) are missense variants located within coding regions, providing a plausible biological mechanism for their role in resistance and highlighting the pipeline’s ability to pinpoint functionally relevant variations. Several SNPs resided in genes with putative roles in stress response or cell wall integrity, such as chitin synthase (AFUA-2G13440) and salicylate hydroxylase (AFUA-7G01050), suggesting novel mechanisms beyond canonical *cyp51A* mutations may contribute to resistance in this population.

Two SNPs (SNP2 and SNP3) showed significant associations (*p* < 0.05) but with a phenotype-specific pattern; SNP2 with itraconazole (ITR) and SNP3 with voriconazole (VOR) (Table 2). Their inclusion is equally important. It demonstrates that our method was not overly restrictive and retained SNPs that may represent secondary resistance mechanisms or alleles with modifier effects. The phenotype-specificity suggests a complex genetic architecture for azole resistance, which would have been overlooked had we used an excessively stringent genome-wide significance threshold at this discovery stage.

### 3.3 Drug resistance phenotypes in sexual offspring

To delineate the genetic effects of the seven candidate SNPs, we strategically allocated them across three distinct crossing pairs of greenhouse-derived strains, with each pair subjected to sexual crossing; subsequently, a total of 300 progeny (100 randomly selected offspring per cross) were evaluated for their minimum inhibitory concentrations (MICs) and growth rates at multiple sub-inhibitory antifungal concentrations (Supplementary Table S5).

**Figure 2:**
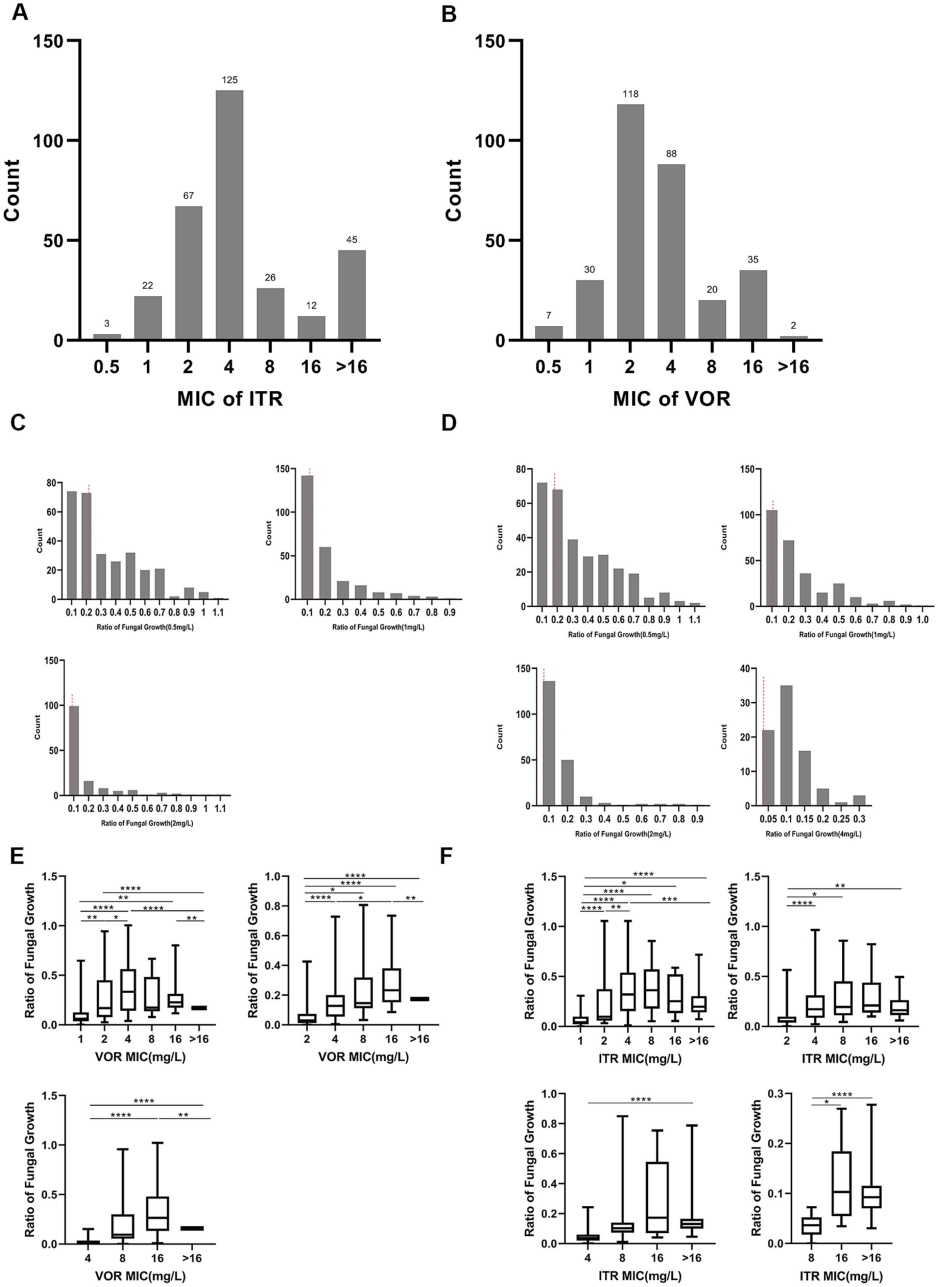
Phenotypic characterization of azole susceptibility in 300 progeny strains of *Aspergillus fumigatus*. **(A)** Distribution of minimum inhibitory concentrations (MICs) for itraconazole (ITR) and **(B)** voriconazole (VOR) among 300 progeny strains. **(C)** Growth ratio distribution of progeny strains under sub-inhibitory concentrations of ITR. Concentrations: (i) 0.5 mg/L, (ii) 1.0 mg/L, (iii) 2.0 mg/L, (iv) 4.0 mg/L. The dashed line indicates the mean growth rate of the parental strains. **(D)** Growth ratio distribution of progeny strains under sub-inhibitory concentrations of VOR. Concentrations: (i) 0.5 mg/L, (ii) 1.0 mg/L, (iii) 2.0 mg/L. The dashed line indicates the mean growth rate of the parental strains. **(E)** Growth rate of 300 progeny strains under different ITR concentrations. (i) 0.50 mg/L, (ii) 1.00 mg/L, (iii) 2.00 mg/L, (iv) 4.00 mg/L. **(F)** Growth rate of 300 progeny strains under different VOR concentrations. (i) 0.50 mg/L, (ii) 1.00 mg/L, (iii) 2.00 mg/L.

Analysis of 300 sexual progeny derived from three independent crosses revealed a broad, continuous distribution of minimum inhibitory concentrations (MICs) for both ITR and VOR, consistent with the polygenic nature of azole resistance. For VOR, 29.33% of progeny (88/300) had an MIC of 4 mg/L, while 11.67% (35/300) and 0.67% (2/300) exhibited higher resistance levels of 16 mg/L and >16 mg/L, respectively. A similar quantitative distribution was observed for ITR, where 41.67% of progeny (125/300) had an MIC of 4 mg/L and 15.00% (45/300) showed high-level resistance (MIC >16 mg/L), as illustrated in Figures 2A,B. This wide phenotypic variation among progeny indicates transgressive segregation, where offspring exceed the phenotypic range of their parents, suggesting that alleles contributing to resistance are recombining in the progeny population.

The growth rates of all 300 progeny and the parental strains were quantified across a gradient of sub-inhibitory ITR and VOR concentrations (0.5, 1.0, 2.0, 4.0, 8.0 and 16.0 mg/L). The growth ratio distribution demonstrated extensive variation at each concentration, with a substantial number of progenies exhibiting growth rates that surpassed or fell below the parental ranges (Figures 2C,D). For instance, in the cross between strains 8–20 and 9–25, 57% of progeny grew better than the parents at 0.25 mg/L ITR, while at 4 mg/L ITR, this proportion decreased to only 8%, indicating a strong selective pressure at higher drug concentrations. The growth ratio distribution of progeny at VOR concentrations of 0.5, 1.0, and 2.0 mg/L also showed substantial variation, though the patterns differed from those observed with ITR (Figure 2C). For example, in the cross 8–20 × 9–25, a high proportion of progeny (78%) grew better than the parents at 0.5 mg/L VOR, suggesting possible different genetic mechanisms underlying resistance to the two azoles (Figure 2D). Meanwhile, 18% of progeny were highly resistant (MIC ≥16 mg/L), even when one parental strain had intermediate resistance (MIC 2–4 mg/L). Offspring exhibited MIC distributions exceeding those of the parents, indicating novel combinations of resistance alleles generated by recombination.

Welch’s *t*-test confirmed significant correlations between MIC values and growth rates across different ITR concentrations for the entire progeny population. At 0.5 mg/L ITR, the growth rate of the low MIC group (1 mg/L) was significantly lower than that of groups with higher MICs (2, 4, 8, 16, and >16 mg/L; *p* < 0.05) (Figure 2E). Significant differences in growth rates between MIC groups were also observed at ITR concentrations of 1.00 mg/L, 2.00 mg/L, and 4.00 mg/L (Figure 2E). Statistical analysis of the 300 progenies confirmed significant differences in growth rates between MIC groups at VOR concentrations of 0.50 mg/L, 1.00 mg/L, and 2.00 mg/L (Figure 2F). The responses were often more pronounced at lower VOR concentrations compared to ITR. Notably, the growth patterns were highly dependent on the specific parental cross, indicating that the genetic background of the parents significantly influences the expression of resistance traits in the offspring. This concentration-dependent response highlights the complex relationship between the standard MIC endpoint and the quantitative growth fitness under drug pressure.

### 3.4 Hierarchical genetic association analysis of azole resistance

This study systematically analyzed the associations of hundreds of genetic models, including single-marker, pairwise marker interactions, and higher-order three-marker interactions, for seven SNP loci with growth rates across two azole drugs (ITR and VOR) under 7 to 13 different concentration gradients.

### 3.5 Genetic background and segregation analysis of progeny population

We first assessed the genetic segregation patterns of seven SNP loci (SNP1-SNP7) in 300 progeny strains derived from controlled crosses of *A. fumigatus* isolates. Chi-square tests revealed that most SNP combinations (e.g., SNP1&2, SNP3&4) followed the expected 1:1:1:1 Mendelian segregation ratio (*p* > 0.05), confirming neutral inheritance and providing a robust population for association analysis. However, combinations involving SNP6 (SNP3&6, SNP4&6, SNP5&6) showed significant deviations from Mendelian expectations (*p* < 0.001), with a strong bias toward the allele inherited from the resistant parent 6–10 (Table 3). This suggests potential selective advantage or linkage disequilibrium at the SNP6 locus, marking it as a key genetic hotspot for further analysis. Building upon this genetic foundation, we constructed multiple higher-order SNP combinations (e.g., SNP3*SNP4&SNP5, SNP4*5*6&3) to probe complex interactions. The genotypic distribution of these combinations not only reinforced the genetic bias at the SNP6 locus but also uncovered diverse allelic recombination patterns (Supplementary Table S6). We subsequently focused on examining how these specific genotypic configurations correlated with fungal growth rates across a spectrum of ITR and VOR concentrations.

**Table 3:** Hierarchical analysis of single SNP combinations: genotype segregation and statistical tests.

Single SNP combination	Parental alleles	Progeny genotype count distribution	Chi-square test (*χ*^2^, *p*-value)	Primary interpretation
SNP1-SNP2	SNP1_8-20/9–25_ & SNP2_8-20/9–25_	SNP1_8-20_&SNP2_8-20_(24) SNP1_8-20_&SNP2_9-25_(27) SNP1_9-25_&SNP2_8-20_(24) SNP1_9-25_&SNP2_9-25_(25)	*χ*^2^ = 0.24, *p* ≈ 0.970	Progeny genotypes fit a 1:1:1:1 Mendelian ratio (no significant bias).
SNP3-SNP4	SNP3_6-10/1–21_ & SNP4_6-10/1–21_	SNP3_6-10_&SNP4_6-10_(21) SNP3_6-10_&SNP4_1-21_(24) SNP3_1-21_&SNP4_6-10_(30) SNP3_1-21_&SNP4_1-21_(25)	*χ*^2^ = 1.68, *p* ≈ 0.641	Progeny genotypes fit a 1:1:1:1 Mendelian ratio (no significant bias).
SNP3-SNP5	SNP3_6-10/1–21_ & SNP5_6-10/1–21_	SNP3_6-10_&SNP5_6-10_(23) SNP3_6-10_&SNP5_1-21_(22) SNP3_1-21_&SNP5_6-10_(28) SNP3_1-21_&SNP5_1-21_(27)	*χ*^2^ = 1.04, *p* ≈ 0.790	Progeny genotypes fit a 1:1:1:1 Mendelian ratio (no significant bias).
SNP3-SNP6	SNP3_6-10/1–21_ & SNP6_6-10/1–21_	SNP3_6-10_&SNP6_6-10_(33) SNP3_6-10_&SNP6_1-21_(12) SNP3_1-21_&SNP6_6-10_(50) SNP3_1-21_&SNP6_1-21_(5)	*χ*^2^ = 50.32, *p* < 0.001	Progeny genotypes fit a 1:1:1:1 Mendelian ratio (no significant bias).
SNP4-SNP5	SNP4_6-10/1–21_ & SNP5_6-10/1–21_	SNP3_6-10_&SNP4_6-10_(45) SNP3_6-10_&SNP4_1-21_(6) SNP3_1-21_&SNP4_6-10_(6) SNP3_1-21_&SNP4_1-21_(43)	*χ*^2^ = 57.84, *p* < 0.001	The progeny genotype distribution did not significantly deviate from the expected 1:1:1:1 Mendelian segregation ratio.
SNP4-SNP6	SNP4_6-10/1–21_ & SNP6_6-10/1–21_	SNP4_6-10_&SNP6_6-10_(48) SNP4_6-10_&SNP6_1-21_(3) SNP4_1-21_&SNP6_6-10_(35) SNP4_1-21_&SNP6_1-21_(14)	*χ*^2^ = 60.2, *p* < 0.001	The progeny genotypes showed a strong bias toward the SNP6 allele inherited from the resistant parent (6-10).
SNP5-SNP6	SNP5_6-10/1–21_ & SNP6_6-10/1–21_	SNP5_6-10_&SNP6_6-10_(47) SNP5_6-10_&SNP6_1-21_(4) SNP5_1-21_&SNP6_6-10_(36) SNP5_1-21_&SNP6_1-21_(13)	*χ*^2^ = 47.6, *p* < 0.001	The progeny genotypes showed a strong bias toward the SNP6 allele inherited from the resistant parent (6-10).

### 3.6 Identification of genetic loci associated with Itraconazole resistance

**Figure 3:**
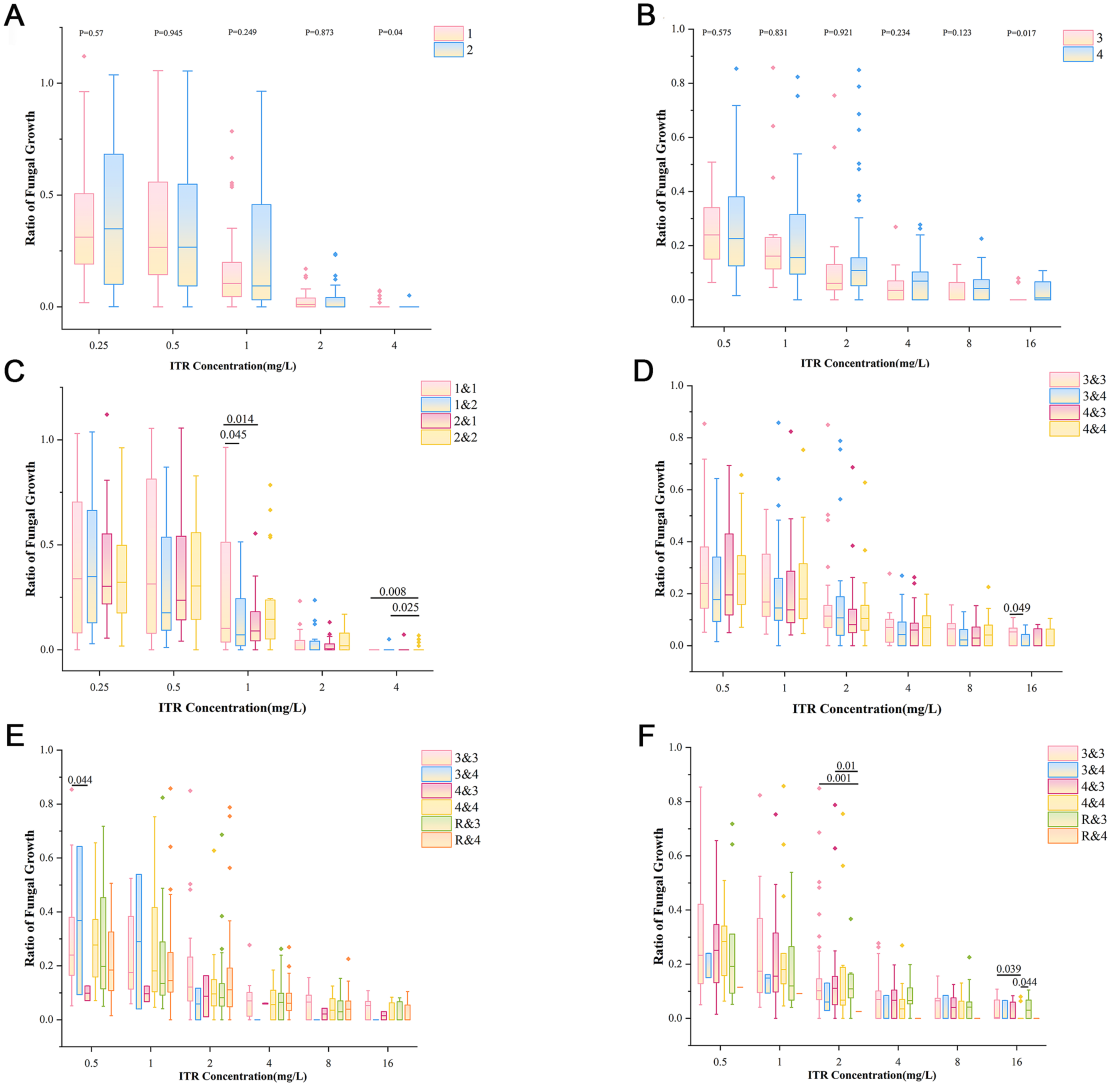
Growth rates of progeny strains under different Itraconazole concentrations, grouped by genotypes at single and combined SNP loci. Growth rates grouped by genotype at single SNP loci: **(A)** SNP1, **(B)** SNP6; Growth rates grouped by genotype at pairwise SNP combinations: **(C)** SNP1&2, **(D)** SNP3&5; Growth rates grouped by genotype at higher-order SNP combinations: **(E)** SNP3 * 4&5, **(F)** SNP4 * 5&6.

Individual SNP analysis under ITR exposure identified SNP1 and SNP6 as significant. At 4 mg/L ITR, SNP1 showed a *p*-value of 0.04, with progeny inheriting the 8–20 allele exhibiting higher growth rates (Figure 3A). SNP6 was significant at 16 mg/L ITR (*p* = 0.017), underscoring its role in high-concentration tolerance (Figure 3B). Other SNPs lacked significant associations, allowing focus on these key loci (Supplementary Figures S3–S6).

Analysis of pairwise combinations revealed epistatic effects. For example, SNP1&2 showed significance at 1 mg/L and 4 mg/L ITR (*p* = 0.045 and *p* = 0.014) (Figure 3C), while SNP3&5 was significant at 0.5 mg/L ITR (*p* = 0.049) (Figure 3D). These interactions demonstrate that ITR resistance involves coordinated actions between loci.

Higher-order combinations, such as SNP3*4&5, showed significance at 0.5 mg/L ITR (*p* = 0.044) (Figure 3E), and SNP4*5&6 was significant at 16 mg/L ITR (*p* = 0.039) (Figure 3F). These results illustrate the polygenic nature of ITR resistance, governed by complex genetic networks.

### 3.7 Identification of genetic loci associated with voriconazole resistance

**Figure 4:**
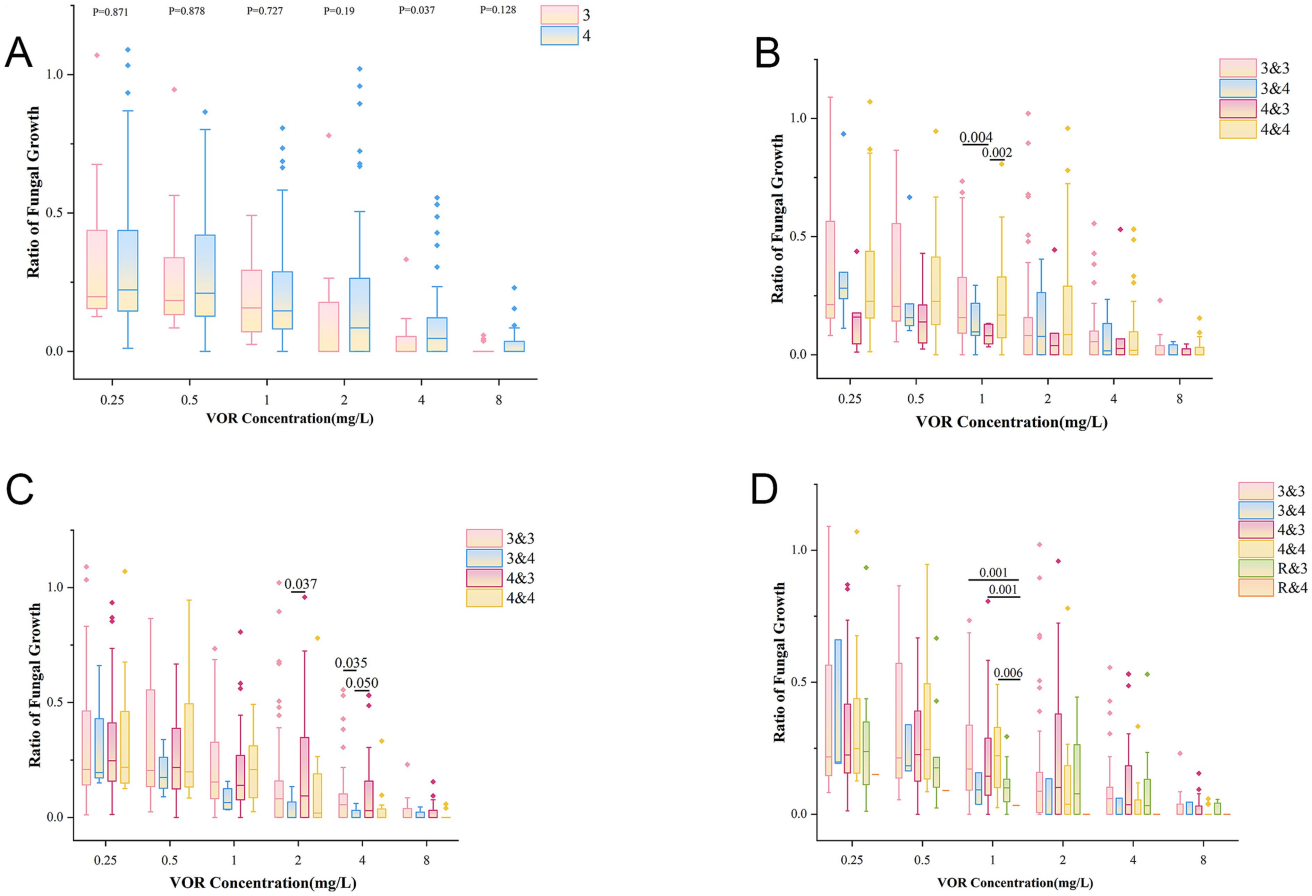
Growth rates of progeny strains under different Voriconazole concentrations, grouped by genotypes at single and combined SNP loci. **(A)** Growth rates grouped by genotype at a single SNP locus: SNP6; Growth rates grouped by genotype at pairwise SNP combinations: **(B)** SNP4&5, **(C)** SNP5&6. **(D)** Growth rates grouped by genotype at a higher-order SNP combination.

For VOR, SNP6 was significant at 4 mg/L (*p* = 0.037) (Figure 4A), but no other single SNPs showed strong associations, indicating greater reliance on multi-locus interactions for VOR resistance compared to ITR.

At 1 mg/L VOR, significant differences in growth ratio were observed between specific genotype combinations. Progeny inheriting the combined heterozygous genotypes exhibited significantly lower growth compared to homozygous parental groups, with *p*-values of 0.004 and 0.002, respectively (Figure 4B), indicating a strong non-additive genetic interaction. This indicates a strong, non-additive genetic interaction between these loci. Another notable pairwise interaction, SNP5&6, was significant at both 2 mg/L and 4 mg/L VOR (*p* = 0.037 and *p* = 0.05) (Figure 4C). Furthermore, higher-order combinations, such as SNP4 * 5&6, were highly significant (*p* < 0.01) (Figure 4D).

Focusing on the significant higher-order combination SNP4*5&6, we observed consistent effects across both drugs. Under VOR at 1 mg/L, progeny with specific allele combinations (e.g., inheriting 6–10 alleles for SNP5 and SNP6) showed significantly higher growth rates (*p* = 0.001) (Figure 4D). This pattern was maintained under ITR, identifying SNP4*5&6 as a robust hub in the resistance network. The absence of significant associations for other SNPs enabled a focused examination of these key loci (Supplementary Figures S7–S10).

The hierarchical association analysis of genetic loci with ITR and VOR resistance revealed a fundamental shared pattern: the prevalence and statistical strength of significant associations increased markedly with the complexity of SNP combinations. Analysis of single SNPs yielded limited significant associations for both drugs (e.g., SNP1 at ITR 4 mg/L, *p* = 0.04; SNP6 at VOR 4 mg/L, *p* = 0.037). In contrast, the examination of pairwise SNP combinations demonstrated a substantial increase in significant interactions (e.g., SNP4&5 for ITR, *p* = 0.002). This trend was profoundly amplified in higher-order combinations, which exhibited a high frequency of highly significant *p*-values (e.g., SNP4*5&6, *p* = 0.001), suggesting that resistance can be largely attributed to complex epistatic networks (Supplementary Table S7).

Within this common framework, parallel analysis also uncovered distinct genetic architectures between the two azoles. SNP6 served as a core locus for both drugs, but exerted stronger single-locus effects in ITR resistance. VOR resistance appeared to rely more heavily on specific multi-locus interactions (e.g., the prominent pairwise interaction of SNP4&5), whereas ITR resistance was characterized by more prominent higher-order networks. Drug-specific effects were evident, such as the significant interaction of the SNP3*4*6&5 combination for VOR but not for ITR, highlighting differential genetic modulation underlying cross-resistance.

## 4 Discussion

### 4.1 Sexual potential retained in resistant greenhouse populations

The discovery of high sexual competency in greenhouse populations, characterized by a balanced mating-type ratio and significantly higher mating success and ascospore viability compared to outdoor strains, significantly advances our understanding of *A. fumigatus* population biology. This finding directly addresses our first research question, showing that high resistance does not compromise sexual potential. The capacity for recombination therefore remains intact, even in populations under intense fungicide selection. While the species was long considered predominantly asexual, the discovery of a fully functional sexual cycle was a paradigm shift (O'Gorman et al., 2009). However, evidence for frequent sexual reproduction in natural, particularly agricultural, settings has remained limited. Our findings align with the hypothesis that Fungi are not strictly asexual replicators; rather, they function as highly environment-sensitive “evolutionary incubators.” When external conditions—such as temperature, light, humidity, or host availability—undergo changes, they trigger the mating program, thereby generating recombined offspring that are better adapted to new ecological niches (Henk et al., 2011; Zhang et al., 2013; Zhang et al., 2022), and we extend this by quantitatively showing that greenhouse environments act as evolutionary incubators. The continuous application of azole fungicides likely imposes strong selective pressure that, directly or indirectly, favors strains capable of genetic recombination, a process that can rapidly assemble advantageous allele combinations (Turo et al., 2021). This contrasts with the clonal expansion patterns often observed for resistant strains in clinical settings (Snelders et al., 2025), suggesting different evolutionary trajectories in distinct ecological niches. The observed sexual compatibility between greenhouse and outdoor populations, albeit at a reduced rate, is a critical finding. It provides a potential genetic conduit for the escape of resistance alleles from agricultural reservoirs into natural populations, a risk that has been hypothesized but for which empirical evidence was scarce (Chowdhary et al., 2013; Sewell et al., 2019). The emergence of isolation-by-distance in outdoor strains further underscores how homogeneous anthropogenic environments can override natural genetic substructuring (Zhou et al., 2023).

### 4.2 Geographic and genetic independence of sexual compatibility

Unlike pathogenic fungi such as *Cryptococcus neoformans*, where mating compatibility often correlates with geographic or genetic proximity (Hitchcock et al., 2025), our results showed that neither factor constrained reproduction in *A. fumigatus,* which aligns with the broader conclusion from Korfanty et al. (2021) that neither geographic nor genetic distance inherently constrains sexual reproduction in *A. fumigatus* at a global scale, while our study extends this understanding by demonstrating that this reproductive independence is maintained even within a localized, anthropogenically pressured greenhouse meta-population, highlighting its resilience to fine-scale spatial and genetic structuring. Successful mating occurred even between genetically divergent or geographically distant strains collected up to 5 km apart. This observation suggests that greenhouse populations form an interconnected reproductive network, enhancing opportunities for allele reshuffling. These findings resolve our second research question, demonstrating that spatial separation does not limit the spread of resistance through sexual recombination.

This pattern of unrestricted mating across genetic and geographic distances within the greenhouse network, however, masks underlying variation in reproductive fitness among subpopulations. Notably, within the interconnected greenhouse meta-population, subpopulation 4 exhibited the highest mating success rate and also harbored the greatest number of alleles among the nine greenhouses, suggesting that high genetic diversity may bolster sexual compatibility. In contrast, greenhouses 6 and 7 showed the lowest mating success, yet possessed the highest numbers of private alleles. This inverse relationship between private allele accumulation and mating success may reflect localized genetic drift or selection leading to reproductive isolation even at fine spatial scales, highlighting how micro-evolutionary processes can differentially shape reproductive traits within a broadly connected population.

Our study reveals a compelling case of population divergence in *A. fumigatus* driven by geographical isolation. The Ninglang population, despite exhibiting the lowest genetic diversity and being the most genetically distinct among all surveyed populations, demonstrated exceptionally high intra-population sexual compatibility. This paradox—high reproductive success internally coupled with significant reproductive isolation from all other geographical populations—strongly suggests that the mountainous terrain surrounding the sampling site has acted as a formidable barrier to gene flow (Zhou et al., 2022; Mitka et al., 2023). This isolation has likely promoted genetic drift and/or local adaptation, making the Ninglang population a striking example of incipient speciation. This finding underscores how geographic barriers can rapidly shape the population structure and reproductive ecology of a ubiquitous fungal pathogen, even on a regional scale.

The high mating success observed in both Subpopulation 4 (with the highest genetic diversity among greenhouses) and the Ninglang outdoor population (with the lowest overall diversity) is not contradictory. Instead, it reveals that different genetic configurations can converge on a similar phenotype of high sexual competency. In greenhouse population 4, a rich and diverse gene pool, particularly of mating-type alleles, likely increases the probability of encountering a compatible partner. Conversely, in the geographically isolated Ninglang population, limited gene flow has led to genetic homogenization and drift. This process may have purged incompatible alleles and fixed a set of co-adapted mating-type loci that function efficiently within the isolated genetic background, thereby ensuring high intra-population compatibility despite low overall diversity. This comparison underscores that high mating success is not a simple function of overall diversity, but rather the specific compatibility of the genetic architecture within a given population context.

### 4.3 Discovery of novel resistance-associated loci

Our GWAS identified 22 SNPs significantly associated with itraconazole and/or voriconazole resistance, all were novel candidates outside *cyp51A*. Previous work has emphasized *cyp51A* tandem repeats and point mutations as the dominant mechanism of azole resistance (Van Rhijn et al., 2021), yet our results reinforce recent studies pointing to additional loci. The inclusion of genes involved in cell wall synthesis, transport, and secondary metabolism indicates that resistance is multifactorial. This directly addresses the third research question by providing genomic evidence for polygenic resistance mechanisms.

The selection of the seven candidate SNPs for progeny-based validation was the result of a deliberate and statistically rigorous bioinformatic strategy, the validity of which is strongly supported by the subsequent phenotypic data from the sexual progeny.

Our multi-tiered filtering approach was designed to balance the discovery of genuine signals with the control of false positives. The initial LD-pruning step ensured that the analysis was based on independent genetic markers, while the subsequent step of expanding the search to include all SNPs in high LD (*R*^2^ > 0.85) with top signals allowed us to focus on genomic loci rather than isolated SNPs, increasing the probability of capturing causal variants. The final application of a stringent Bonferroni correction within this focused candidate set served as a robust statistical gatekeeper.

The effectiveness of this strategy is compellingly demonstrated by the spectrum of association strengths observed among the seven selected SNPs. The results form a biologically interpretable gradient: the majority of the candidates (five out of seven, including SNP1, SNP4, SNP5, SNP6, and SNP7) exhibited exceptionally strong associations with azole resistance (*p*-values ranging from 10^−5^ to 10^−4^), confirming that our pipeline successfully enriched for SNPs with major phenotypic effects. Notably, some of these (e.g., SNP4, SNP5) are missense variants, suggesting a direct functional consequence. The remaining two SNPs (SNP2 and SNP3) showed significant, albeit weaker and more compound-specific, associations. This is not a weakness of the method but rather a strength; it demonstrates that the approach was not overly restrictive and retained SNPs that may represent secondary resistance mechanisms or modifier genes, reflecting the polygenic nature of the trait. Crucially, the complete absence of non-significant SNPs among the final seven candidates underscores the high specificity of our filtering cascade. Therefore, we argue that the seven candidate SNPs represent a high-confidence set of genetic targets, provides a solid foundation for the conclusion that azole resistance in these populations is influenced by a complex network of interacting genetic loci.

### 4.4 Functional implications of genetic associations and interactions of seven SNP loci in azole resistance

Sexual offspring displayed MIC distributions that exceeded those of the parents, including 18% with high-level resistance (MIC ≥16 mg/L). This result highlights how recombination can produce resistant genotypes not observed in parental strains, a phenomenon also noted in experimental crosses (Korfanty et al., 2021). Furthermore, fitness assays revealed variable costs associated with resistance, with some offspring maintaining high resistance without substantial growth penalties. These findings emphasize the evolutionary potential of sexual reproduction to generate novel adaptive genotypes in resistant populations.

At the genetic level, our hierarchical association analysis reveals that azole resistance is a polygenic trait, with the data suggesting that its genetic architecture is largely epistatic in nature. The most striking pattern is the dramatic increase in the strength and prevalence of significant associations as SNP combinations increase in complexity. This provides strong, direct evidence that the phenotypic outcome is determined by specific allelic configurations within interactive genetic networks, a concept often proposed but difficult to demonstrate in fungal pathogens (Sun et al., 2022). While seminal work has justifiably focused on gain-of-function mutations in the *cyp51A* gene, our GWAS approach, focused on a population where non-*cyp51A* mechanisms are prevalent (Zhou et al., 2021), successfully identified novel genetic loci. The enrichment of associated SNPs in genes involved in stress response and cell wall integrity provides a genetic basis for the observed epistatic networks. This points to plausible biological mechanisms—such as coordinated drug efflux, cell wall remodeling, or activation of compensatory metabolic pathways—that are known to operate in other fungal systems (Yan et al., 2025).

Our hierarchical genetic analysis, integrating association mapping with functional annotations of the seven candidate SNP loci, reveals a complex, epistatic network underpinning azole resistance in *A. fumigatus*. The center of this association network is defined by SNP6 (AFUA_8G00342), which emerged as a central node, demonstrating significant single-locus effects and recurring in critical higher-order interactions (e.g., SNP4 * 5&6). While its molecular function remains uncharacterized, its consistent and strong association with resistance across multiple contexts marks it as a promising genetic factor in adaptive evolution, warranting future functional characterization.

The roles of other annotated SNPs contextualize the polygenic nature of resistance within specific cellular pathways. The involvement of SNP4, encoding a salicylate hydroxylase linked to oxidative stress response, is particularly instructive (Costa et al., 2019). Its strong association in VOR-specific interactions (e.g., SNP4&5) aligns with the distinct genetic architecture observed for VOR resistance and supports the hypothesis that VOR tolerance may rely heavily on pathways mitigating drug-induced oxidative damage. The contributions of SNP1 (pyrimidine 5′-nucleotidase, potentially involved in metabolic stress response), SNP2 (dynamin-family GTPase, implicated in membrane trafficking), and SNP7 (chitin synthase ChsE, crucial for cell wall integrity) further illustrate how diverse biological processes—from nucleotide metabolism and vesicle dynamics to structural maintenance—are co-opted into the resistance network through epistatic interactions (Rothman et al., 1990; Barthel et al., 2024; Nakanishi and Sekimizu, 2002). The phenomenon of transgressive segregation in progeny, a hallmark of quantitative traits, is directly explained by the recombination of these functionally diverse, epistatic loci during meiosis (Steensels et al., 2021; Samarasinghe et al., 2020).

Our parallel analysis of ITR and VOR resistance further clarifies how this shared genetic toolkit is configured differently for each drug. The fact that VOR resistance demonstrated a greater reliance on specific pairwise interactions (like the prominent SNP4&5), whereas ITR resistance involved more prominent higher-order networks, likely reflects differences in the chemical properties and cellular stressors imposed by each triazole. This nuanced view adds depth to the understanding of cross-resistance and is consistent with the idea that fungi employ distinct genetic strategies to cope with different azole compounds (Hagiwara et al., 2018). The drug-specific significance of combinations like SNP3*4*6&5 further underscores that cross-resistance is not a uniform trait but is differentially modulated by genetic context.

Building on our findings, we suggest a synergistic model for the rapid evolution of resistance in agricultural settings, in which high basal sexual competency might facilitate recombination, acting on a standing genetic landscape shaped by epistatic interactions (potentially involving loci like SNP6 and SNP4). This perspective integrates concepts of sexual reproduction as an adaptive tool with the quantitative genetics of complex traits, providing a testable framework for understanding rapid pathogen adaptation (McDonald and Linde, 2002). Our results suggest possible implications: the concentration-dependent SNP effects imply that sub-inhibitory drug exposure could influence the selection of genetic variants, which may be relevant for dosing strategies. Moreover, the evidence for epistasis highlights the potential importance of considering genetic backgrounds in resistance surveillance. Future research could prioritize functional characterization of key nodes (e.g., SNP6) and mapping of the full epistatic network to better understand and mitigate resistance evolution.

## 5 Conclusion

This study provides a multi-faceted understanding of how azole resistance evolves in agricultural populations of *A. fumigatus*. We first established that greenhouse populations function as hubs of high sexual fitness, characterized by significantly greater mating success and ascospore viability compared to outdoor strains. Second, the strength of genetic associations increased substantially with the complexity of SNP combinations, underscoring that resistance arises from synergistic interactions within genetic networks, not from the additive effects of single loci. Furthermore, we uncovered that the genetic underpinnings of resistance to the two azoles are distinct: voriconazole resistance relies more on specific pairwise interactions, whereas itraconazole resistance involves broader, higher-order epistatic networks.

Thus, we propose a synergistic model for rapid adaptation: the high sexual competency of these populations provides a platform for generating genetic novelty through recombination, while the pre-existing, complex epistatic landscape serves as the substrate upon which azole selection can act efficiently. This work highlights agricultural environments as critical arenas for resistance evolution and underscores the necessity of expanding surveillance beyond single mutations to include the multi-locus genetic backgrounds that ultimately determine the resistant phenotype. Future research should prioritize the functional characterization of key hubs like SNP6 to identify new targets for intervention.

While this study focused on the genetic architecture of resistance, future investigations into how specific biotic (e.g., microbial community) and abiotic (e.g., pesticide use, humidity) factors within greenhouse environments may have shaped the observed genetic variation and selection for these epistatic networks would provide a more comprehensive ecological-evolutionary perspective.

## Data Availability

Raw FASTQ files for the isolates sequenced in this study were uploaded to the NCBI Sequence Read Archive (SRP637305) and are publicly available under BioProject PRJNA1345449.
