# Supplementary material for: Sexual reproduction and the polygenic architecture of azole resistance in agricultural populations of Aspergillus fumigatus
Source: Front Microbiol. 2026 Jun 3;17:1843477. doi: 10.3389/fmicb.2026.1843477 (PMC13274624; doi:10.3389/fmicb.2026.1843477)
Supplement: Supplementary file 3 [file Table_3.DOCX]

Table S2. Primer sequences used for genotyping the seven candidate SNP loci.

| Primer Name | Sequence (5' → 3') |
| --- | --- |
| SNP1-L | 5′-GTCCCTTCCTTCTCTACA-3′ |
| SNP1-R | 5′-AAAGCCAACGAAACTATT-3′ |
| SNP2-L | 5′-AGCGGGCTCGTGGTATC-3′ |
| SNP2-R | 5′-AAGGCGGCTGGTTTATTC-3′ |
| SNP3-L | 5′-GACGCCTCGGTTCTCATT-3′ |
| SNP3-R | 5′-TGTTCTCCTCGGCTCTGT-3′ |
| SNP4-L | 5′-TGAAGAATGGCGAGTTTG-3′ |
| SNP4-R | 5′-AGTACGGGTGCTTGAGGG-3′ |
| SNP5-L | 5′-GTTCACTTCAATACTACG-3′ |
| SNP5-R | 5′-CTCTCTGCTGATGCTACA-3′ |
| SNP6-L | 5′-TTCTCGTCTTCACAAATA-3′ |
| SNP6-R | 5′-AAACAAAGTACCCACAGC-3′ |
| SNP7-L | 5′-CTCTTCTAGTGTCTTTAATAACAGG-3′ |
| SNP7-R | 5′-GCCTACCTTTAAGAGATGCATAG-3′ |
